# Farm size limits agriculture's poverty reduction potential in Eastern India even with irrigation-led intensification

**DOI:** 10.1016/j.agsy.2023.103618

**Published:** 2023-04

**Authors:** Anton Urfels, Kai Mausch, Dave Harris, Andrew J. McDonald, Avinash Kishore, Balwinder-Singh, Gerardo van Halsema, Paul C. Struik, Peter Craufurd, Timothy Foster, Vartika Singh, Timothy J. Krupnik

**Affiliations:** a International Maize and Wheat Improvement Centre (CIMMYT), Sustainable Agrifood Systems Program (SAS), Nepal; b Water Resources Management Group, Wageningen University & Research, Netherlands; c Centre for Crop Systems Analysis, Wageningen University & Research, Netherlands; d World Agroforestry (ICRAF), Nairobi, Kenya; e School of Natural Sciences, Thoday Building, Bangor University, Bangor, Gwynedd LL57 2UW, UK; f International Crops Research Institute for the Semi-Arid Tropics (ICRISAT), Nairobi, Kenya; g Section of Soil and Crop Sciences, School of Integrative Plant Sciences, Cornell University, New York, USA; h South Asia Office, International Food Policy Research Institute, New Delhi, India; i International Maize and Wheat Improvement Centre, Sustainable Agrifood System Program (SAS), India; j School of Mechanical, Aerospace and Civil Engineering, University of Manchester, United Kingdom; k International Maize and Wheat Improvement Centre (CIMMYT), Sustainable Agrifood Systems Program (SAS), Bangladesh; l Department of Primary Industries and Regional Development, Northam, Western Australia, Australia

**Keywords:** South Asia, Intensification Benefit Index, Groundwater, Sustainable agriculture, Rural transformation, Indo-Gangetic Plains, Rice-wheat system

## Abstract

**CONTEXT:**

Millions of people living in the Eastern Gangetic Plains (EGP) of India engage in agriculture to support their livelihoods yet are income poor, and food and climate insecure. To address these challenges, policymakers and development programs invest in irrigation-led agricultural intensification. However, the evidence for agricultural intensification to lift farmers' incomes above the poverty line remains largely anecdotal.

**OBJECTIVE:**

The main objective of this study is to use a large household survey (*n* = 15,572; rice: 8244, wheat: 7328; 2017/18) to assess the link between agricultural intensification and personal daily incomes from farming (FPDI) in the rice-wheat systems of the EGP – the dominant cropping system of the region.

**METHODS:**

We use the Intensification Benefit Index (IBI), a measure that relates farm size and household size to FPDI, to assess how daily incomes from rice-wheat production change with irrigation-led intensification across the EGP.

**RESULTS AND CONCLUSIONS:**

Relative to the international poverty line of 1.90 Purchasing Power Parity (PPP)$ day^−1^ and accounting for variations in HH size in the analysis, we found that small farm sizes limit the potential for agricultural intensification from irrigation to transform the poverty status of households in the bottom three quartiles of the IBI. The estimated median FPDI of households with intensified systems in the bottom three quartiles is only 0.51 PPP$ day^−1^ (a 0.15 PPP$ gain). The median FPDI increases to 2.10 PPP$ day^−1^ for households in the upper quartile of the IBI distribution (a 0.30 PPP$ gain). Irrigation-led agricultural intensification of rice-wheat systems in the EGP may provide substantial benefits for resilience to climatic change and food security but achieving meaningful poverty reduction will require complementary investments.

**SIGNIFICANCE:**

Transforming the poverty status of most smallholder farmers in the EGP requires diversified portfolios of rural on- and off-farm income-generating opportunities. While bolstering food- and climate security, agronomic intervention programs should consider smallholders' limited monetary incentives to invest in intensification. Irrigation-led agricultural intensification programs and policies should explicitly account for the heterogeneity in household resources, irrigation levels, and degree of dependence on agricultural income.

## Highlights

• Irrigation is crucial for food security and climate resilience
• We assess the link between agricultural intensification of smallholder farms and personal daily incomes from farming
• Rice-wheat production can only lift large farms above the poverty line
• Reducing farmer poverty needs multi-sectoral investments and off-farm jobs
• Targeted investments are required to strengthen capacity for staple production

## Graphical abstract

**Figure:**
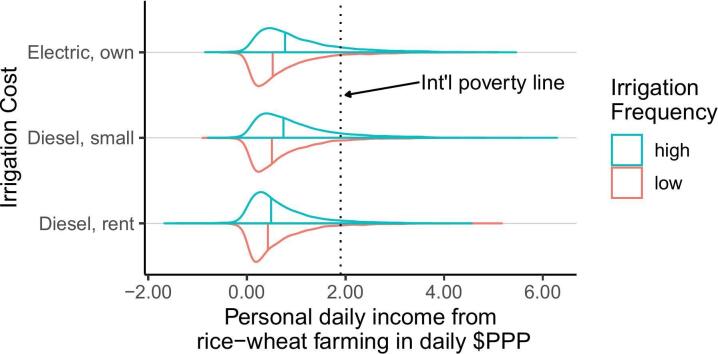

## 1 Introduction

Agricultural intensification and enhanced resilience to water stress through irrigation development is a widely discussed approach for achieving food security (Sustainable Development Goal (SDG)2), climate action (SDG13), and poverty reduction (SDG1) in smallholder-dominated poverty hotspots such as the Eastern Gangetic Plains (EGP) of South Asia. From 1994 to 2012, poverty in the Indian state of Bihar, which encompasses a large part of the EGP, has been reduced from 61% to 34%. This figure still lags behind national averages in the region such as 21% in India, 15% in Nepal and 20% in Bangladesh as of 2010 (World Bank Group, 2016). Situated between the Himalayas and the Bay of Bengal, agricultural production risks are increasing in the Eastern Gangetic Plains due to a progressively more erratic monsoon cycle and high exposure to climate shocks such as droughts and heat (Sheth, 2015). To adapt to increasing dry spells, groundwater is the main source of supplemental irrigation water for farmers in the EGP, but reliable access and associated irrigation intensities vary widely (Foster et al., 2019; Shah et al., 2009; Urfels et al., 2020). Consequently, policy initiatives in the Indian EGP promise to transform agriculture by doubling farmers' incomes through irrigation-led agricultural intensification that relies on expanding groundwater use (Lele, 2019; Struik and Kuyper, 2017). These initiatives focus on investments in better irrigation infrastructure, entrepreneurship, and irrigation services to reduce climate risks and increase agricultural productivity.

However, while there is ample literature on the potential of irrigation-led agricultural intensification to increase yields (especially under controlled conditions), the potential for directly reducing poverty by raising farmer incomes from crop production is poorly understood. As highlighted by Balasubramanya and Stifel (2020), the evidence on linkages between irrigation and poverty reduction remain limited although previous research has outlined the importance of cross-sectoral and indirect effects of irrigation development on poverty reduction (Namara et al., 2010). More recently, studies in Sub-Saharan Africa (Frelat et al., 2016; Harris, 2019) have shown that investing in agricultural production may only provide modest improvements in households' poverty status and increasingly cross-sectoral efforts are needed to reach SDG1. These studies showed that land per capita ratios limit the personal daily incomes from farming (FPDI) that can be expected from agricultural intensification when compared to national and international poverty lines (Harris, 2019). However, such evidence remains scarce in the rice-wheat systems of the EGP and filling this knowledge gap may provide critical insights for designing targeted policies and development programs for the sustainable intensification of agriculture.

In this paper, we use the Intensification Benefit Index (IBI) (Harris, 2019) and a unique large *n* dataset to assess the opportunity space for irrigation-led agricultural intensification to increase the income farmers can derive from rice-wheat production systems in the EGP. We evaluate these gains vis-à-vis the international poverty line of Purchasing Power Parity (PPP)$1.90 day^−1^ (World Bank, 2020). We find that while irrigation improves crop yields, small farm sizes limit the income increases farmers may gain through agricultural intensification. This paper investigates four aspects: First, we explore the distribution of households' IBI values (akin to a household's land per capita ratio) to understand farm sizes and their impact on daily incomes from crop production. Second, we compare the productivity of rice-wheat production and conservatively assess production costs to benchmark households' FPDIs and calorie provisioning associated with increasing numbers of irrigation applications. These estimates assume free irrigation. Third, we conduct a sensitivity analysis around the cost of irrigation to explore the impact of varying irrigation prices associated with different irrigation technologies on our estimated FPDI values. Fourth, we assess trends in home consumption and market participation patterns of irrigated rice-wheat production and their implications for the overall livelihoods of farming households.

## 2 Materials and methods

### 2.1 Study area & data

The EGP encompasses parts of the Indian states of Uttar Pradesh and Bihar, the Terai region of Nepal, and northwestern Bangladesh and contrasts with the drier Middle and Upper Gangetic Plains in Western India and Pakistan. The region generally receives between 1000 and 1500 mm of rainfall per year, of which >80% occurs in the monsoon months June–September. The soils and associated aquifers represent some of the world's most extensive alluvial plains formed by the meandering Ganges and its tributaries that carry sediments from the Himalayas. Smallholders, farmers working on ≤10 ha of land and within our sample not exceeding 5 ha (see Table 1), predominantly grow rice (>90%) in the monsoon season followed by mainly wheat (>60%) but also other crops such as lentils, oilseeds, or potatoes that are planted on residual moisture after the rice harvest in November and are harvested in late March.

**Table 1:** Overview of descriptive summary statistics for key variables. Landholding, irrigation frequency, fertilizer cost, and yield are raw input data. Other variables were calculated for each household as described in the Methods section. Source: Household Data (see Section 2.1 for details).

		Landholding size in ha	Number of household members	Irrigation Frequency	Fertilizer cost in PPP$ ha^−1^ season^−1^	Yield in t ha^-1^	Intensification Benefit Index in cents dollar^−1^	Personal daily income in PPP$ day^−1^	Profit in PPP$ ha^−1^ year^−1^
Rice (*n* = 8244)	Mean	0.85	7.91	3.90	270	4.06	0.031	0.59	1720
	SD	0.76	2.66	2.42	154	1.19	0.029	0.71	1045
	Min	0.01	1.00	0.00	0	0.52	0.000	−1.14	−3860
	Q1	0.33	6.00	2.00	167	3.25	0.012	0.13	996
	Median	0.63	8.00	3.00	242	4.00	0.022	0.36	1688
	Q3	1.10	10.00	5.00	358	4.80	0.041	0.78	2435
	Max	4.85	12.00	13.00	5114	13.54	0.196	4.72	4455
Wheat (*n* = 7328)	Mean	0.69	7.92	2.27	342	2.98	0.025	0.32	1210
	SD	0.68	2.71	0.76	92	0.84	0.025	0.44	708
	Min	0.01	1.00	1.00	0	0.53	0.000	−1.03	−1119
	Q1	0.25	6.00	2.00	285	2.40	0.009	0.08	687
	Median	0.49	8.00	2.00	346	3.00	0.017	0.18	1161
	Q3	0.80	10.00	3.00	409	3.40	0.031	0.39	1629
	Max	4.98	12.00	5.00	794	6.50	0.196	4.24	4109

**Fig. 1:**
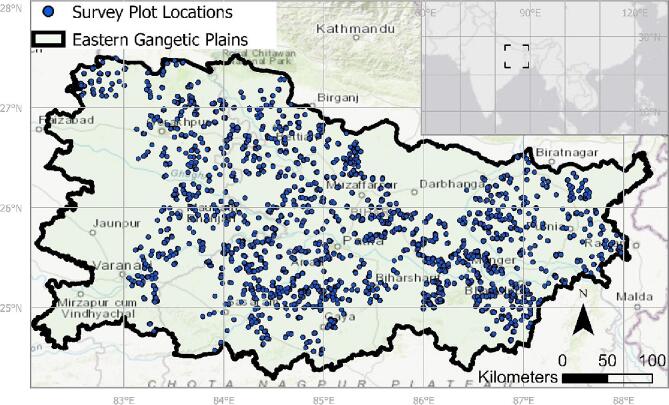
Map of study location and survey data points.

Household-level production data for farmers' main rice and wheat plots in 2017–2018 (henceforth ‘household data’) were collected with an ODK-assisted questionnaire as part of a collaborative data collection effort between the Cereal Systems Initiative for South Asia (www.csisa.org) and the Indian Council of Agricultural Research (ICAR) (for details, see e.g., Ajay et al., 2022). Key modules included landholding characteristics, plot characteristics, input and management activities, and yield outcomes. Data was collected from 10 randomly selected households from 25 randomly selected villages across 36 districts in Eastern Uttar Pradesh and Bihar. For each season, households were sampled independently. This led to a total of 18,000 household – cropping season observations of which 16,016 were retained after quality control (see Fig. 1).

We analyzed the household survey data from the 2017–2018 rice-wheat-rice season in the EGP with the following crop-year combination: rice-wheat = 16,016; thereof rice: *n* = 8589 and wheat: *n* = 7427. Among wheat farmers, 81% grew rice before wheat, while 75% of rice farmers grew wheat as the previous crop in 2017 and 84% in 2018. The second largest category of previous crops grown was ‘fallow’ for both rice and wheat. Rabi rice was not considered in this study. Land fragmentation poses challenges to collecting production data from smallholder environments as management may vary from plot to plot. We simplified the analysis by assuming that farmers applied the same management practices and obtained the same yields as on their largest plot across all plots (Fraval et al., 2019; Niroula and Thapa, 2005). Furthermore, as is common with similar datasets, farmers' inaccurate estimates of landholding and very small plot sizes can cause large positive outliers, which we removed by consecutively trimming off households in the 99th percentile of affected variables (i.e., IBI, FPDI, landholding size, and profits). This procedure resulted in a total dataset of 15,572 records (rice: 8244; wheat: 7328). Demographically, educational status of respondents shows that the sample reflects a diversity of backgrounds: masters (1.6%), bachelors (8.7%), secondary school (11%), primary school (30%), matriculation (21%), no schooling (27%).

Precipitation was average for the rice season 2017, with a Standardized Precipitation Index (SPI) ∼0, and below average for the wheat 2018 and rice 2018 season, SPI ∼ −1 to −3 (IRI, 2020). This means that our data only partially account for weather factors and therefore can only offer limited inference regarding resilience and robustness provided by increased irrigation intensities to the rice-wheat systems of the EGP. Specifically, the data offer ‘high side’ estimates of irrigation advantages due to prevailing climate conditions.

### 2.2 Profit estimation for fully subsidized irrigation and intensification benefit index

#### 2.2.1 Intensification benefit index

To analyze the effect of increased use of irrigation on household incomes, we first calculated the Intensification Benefit Index (IBI) of households (Harris, 2019). The IBI indicates what a farm household earns in dollar per day terms (US$/person/day) from generic farm system profitability values expressed in dollar returns per ha per year terms (US$/ha/year) and is a function of farm size and household size (Eq. (1)). Since both parts of this ratio are expressed in the same currency, IBI may be used to compare directly farming households in different countries in a unit of cents/dollar. IBI is proportional to household land per capita (LPC) and Eq. (1) simplifies to LPC/365.

(1) $$\mathrm{IBI}\;\left(\frac{\mathrm{cents}}{\mathrm{dollar}}\right)=\frac{\left(\frac{1\;\text{\$}/\mathrm{ha}/\mathrm{year}*100}{365\;\mathrm{days}}\right)}{\mathrm{household size}\;\left(\mathrm{persons}\right)\;}*\mathrm{cropped area}\;\left(\mathrm{ha}\right)$$

#### 2.2.2 Personal daily incomes from farming (FPDI)

We estimated FPDI in US$ day^−1^ from the annual profitability values per hectare reported for rice and wheat production by households for each observation (Eq. (2)). To allow for international comparisons of income measures and comparison against the international poverty line, we converted the input and sales costs to PPP$ by using a conversion factor of 18.10 INR-PPP$ as reported by the World Bank for 2018. Subsequently, we calculated the value of total production by multiplying self-reported yields in t/ha with the reported farm gate price in (PPP$ t^−1^). We treated the full net value of production as income since farmers would have to purchase grains for a similar price if home consumption were absent, thus neglecting factors such as additional costs associated with commercial value chains, price fluctuations in time, and quality differences to maintain parsimonious analysis. We also used the IBI to calculate the crop-specific personal daily calories available (in kcal day^−1^) from total production per ha using an average value of 2800 kcal kg^−1^ for rice and 3340 kcal kg^−1^ for wheat (D'Odorico et al., 2014).

(2) $$\mathrm{FPDI}=\left[\left(\mathrm{yield}*\mathrm{farm gate price}\;\right)-\mathrm{input cost}\;\right]*\mathrm{IBI}$$

We then approximated profits by subtracting input costs in PPP$ ha^−1^. Since our dataset does not contain full cost of production information, we approximated FDPIs by using key cost of production values (machinery, seed, labor, and fertilizer). For fertilizer, we multiplied the amounts of fertilizers that individual surveyed farmers reported to have applied with its typical costs per kg (Urea PPP$ 0.9; DAP 1.06 PPP$). For the remainder we used values as reported by the Indian Government for the state of Bihar for machinery (rice: 221.82 $PPP ha^−1^, wheat: 327.96 $PPP ha^−1^), seed (rice: 178.67 $PPP ha^−1^, wheat: 178.67 INR ha^−1^), and hired labor (wheat: 251.05 $PPP ha^−1^, rice: 525.80 $PPP ha^−1^) as our dataset did not include this information (CACP: Cost of Cultivation Report 2017) (Commission for Agricultural Costs and Prices, 2017a, Commission for Agricultural Costs and Prices, 2017b). For irrigation cost, we first treated irrigation as free (i.e., fully subsidized) and then conducted a sensitivity analysis that accounts for the different types of typical irrigation systems and associated costs as described at the end of this section. In addition, we compared the FPDIs that account for the net value of production with cash incomes by multiplying FPDIs with the self-reported marketed share of production and explored daily calories per capita retained by households. We further present the self-reported share of agricultural income in total household income as well as the surveyed crop's share of agricultural income.

#### 2.2.3 Irrigation, yield, and daily incomes

We assessed how yields and daily incomes differ across the rainfed-fully irrigated spectrum. Here, our goal was not to isolate the causal effect of irrigation frequency on yields and daily incomes, but to identify realistic yield and daily incomes that farmers may obtain for different levels of irrigation intensity based on the observations in our dataset. Given the limitations of our costs data, we further ensure the robustness of our results by contextualizing our findings through investigations of gross value of production, profit ha^−1^ values from the literature and relating these two to the IBI values of the farms in our dataset. We then contrasted the profitability of the rice-wheat system for farms of low and high irrigation intensities by separating households into groups of low and high irrigation based on the range of irrigation intensities observed in the region (see Table 1). That is, < 3 irrigations in rice (28%) and wheat (63%) each for the low group and >3 irrigations in rice (44%) and >2 irrigations in wheat (36%) for the high irrigation group. Due to the lack of panel data, we summed the rice and wheat distributions for each group to assess the overall system benefits. We further present results of a random forest model that serves to explore average trends and variability in yields and daily profits associated with different irrigation frequencies in our dataset (Biau and Scornet, 2016; Breiman, 2001).

As the relationship between irrigation intensity and daily incomes is – in theory – non-linear, we used a non-parametric random forest model to estimate the shape of average yield and daily incomes for different irrigation frequencies within our dataset (Hastie and Tibshirani, 1990; Roberts, 2004; Yee and Mitchell, 1991). Various factors (e.g. soil types, varieties, fertilizer rates etc.) may influence how farms with lower irrigation frequencies respond to increasing their levels of irrigation. Nevertheless, due to its large size our dataset covers many variations across these factors including across irrigation frequencies. The average yield and income values for high irrigation frequencies thus serves as a reasonable baseline for these systems. Given that our dataset does contain some of the key contributing factors, we investigated their impact by including them in the random forest regressions to calculate partial dependency plots (Lundberg and Lee, 2017; Yang, 2021) that account for a large number of predictors including soil and drainage class, education, fertilizer rate, share of crops sold, landholding size, weeding times, abiotic and biotic stress occurrence, crop duration, market distance, variety type, planting date, plot ownership, timeliness of input availability, market distance, and irrigation sources. The yield prediction model was fit with the fast ranger implementation of random forest in R (Wright and Ziegler, 2017) to overcome performance issues with the original implementation (Liaw and Wiener, 2002). The model was run with 500 trees and mtry set to the square root of the number of variables.

This model showed that benefits of irrigation varied regionally and with other factors and co-variates such as soil type, crop types, input intensity, and farmers' education (which lie outside the scope of this paper). However, as expected due to the large size of the dataset, the sign and magnitude of yield and income levels between rainfed and fully irrigated systems were confirmed even when other factors and covariates were accounted for (see Section 3.2). Importantly, farm size remained the major governing variable for daily incomes. In addition, studies regarding the efficiencies and farm size – productivity dynamics among small farms (Deininger et al., 2017; Paul and Githinji, 2018), indicate a certain degree of endogeneity may affect causal inference analytics on the impact of irrigation on farm incomes and need to be carefully considered in future studies. In the Supplementary materials, we provide exploratory overviews of (i) partial effects estimates of GAM models for irrigation frequency and FDPIs across IBI groups and (ii) smoothing splines for estimated FDPI across irrigation levels for different crop type and soil type combinations to provide interested readers further details.

Lastly, for the sensitivity analysis of irrigation costs, we assessed how the profitability of irrigation-based intensification changed with typical pumping costs. We used irrigation cost values based on fieldwork data and secondary literature and included (rented) large diesel pumps, small diesel pumps, (rented) electric pumps, and fully subsidized irrigation (Foster et al., 2019; Shah et al., 2009; Urfels et al., 2020). We assumed an irrigation of 60 mm, 3.59 $PPP/l of fuel, 1.21 $PPP/unit of electricity; Large pumps: 1.25 l/h fuel consumption and 12 l/s discharge; Small pumps: 0.5 l/h fuel consumption and 10 l/s discharge; Electric: 1 unit/h energy consumption and 8 l/s discharge.

## 3 Results and discussion

### 3.1 Intensification Benefit Index distribution and the median household

The households in our dataset had a very small median IBI value of 0.02 cents per dollar^−1^ with a strongly right-skewed distribution (see Table 1 for crop-wise figures). This means that a household with the median IBI value with crop production profits of PPP$ 1000 ha^−1^ year^−1^ would earn PPP$ 0.20 day^−1^. A profit of 9500 PPP$ ha^−1^ year^−1^ would be required to provide the PPP$1.90 day^−1^ needed to move above the international poverty line (World Bank, 2017). The median number of household members in our dataset was 7.8 with 0.54 ha for a household's landholding (see Table 1 for crop-wise figures). To contextualize, the median IBI value of 0.02 cents dollar^−1^ may represent a household with 0.64 ha of land and 8 persons, 0.32 ha of land and 4 persons or, more generally, a land per capita ratio of 0.08 ha person^−1^.

To understand the IBI logic, consider the following thought experiments: If the landholding of a household would increase (e.g., double due to land purchases) the cropping system profitability requirements for reaching the poverty line would be cut in half (4500 PPP$ ha^−1^ year^−1^). Similarly, as the number of household members decreases, IBI values increase as relatively more land is available per person. For example, if a household member left the household (e.g., young adults to pursue work opportunities elsewhere) this would also decrease the profit requirements to lift the farming household above the poverty line (but may impose additional labor costs that might constrain achievement of that profit).

In addition, productivity and management of small farms might be further constrained by other factors such as machinery and labor availability even if irrigation intensity is increased (Urfels et al., 2021). Consequently, irrigation-led intensification is likely to benefit from delineating areas where substantial accompanying investments are required to lift other production constraints before the benefits of irrigation could materialize.

**Fig. 2:**
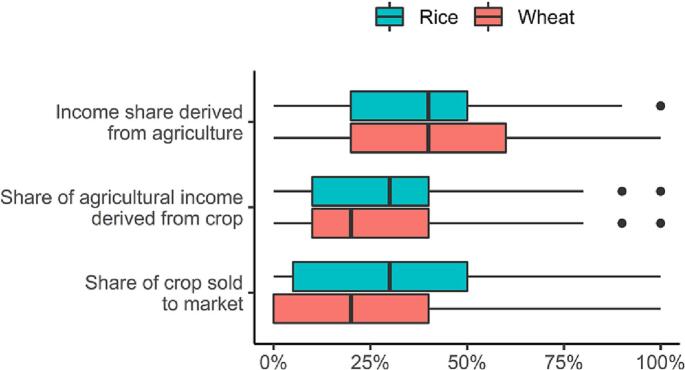
Distributions of income shares and marketed shares of crops for rice-wheat farmers in the EGP.

Furthermore, we found that for the median rice + wheat growing household in our sample the estimated full net value of production amounted to PPP$ 2905 ha^−1^ year^−1^ and PPP$ 0.56 day^−1^ (see Table 1 for crop-wise figures). Average yields of 3.9 t/ ha (rice) and 2.8 t/ ha (wheat) provided 4054 kcal person^−1^ day^−1^ at the median IBI of 0.02 (see Table 1). While not lifting households above the poverty line, rice-wheat systems provide important contributions to household food security. Accordingly, our data shows that most of the production is consumed rather than sold (median sold share of income: PPP$ 572 ha^−1^ year^−1^ and PPP$ 0.11 day^−1^ (see Section 3.4). In addition, farmers tended to complement farm incomes with off-farm income sources. For the median household, incomes from rice-wheat accounted for only 20% of total income, and agriculture, in general, accounted for ca. 40% (see Fig. 2 and Section 3.4). That is, the median household earned ca. PPP$ 0.55 day^−1^ from sources other than rice-wheat production. Together, these figures suggest that rice-wheat production contributed a considerable share to household food security but, although it decreases the depth of poverty, a substantial increase in incomes from crop production would be required to lift the median household above the poverty line.

Altogether, our data indicates that most farmers in our dataset live well below the international poverty line with rice-wheat production being worth PPP$ 0.56 or less for 50% of the population (increasing to PPP$ 1.11 when considering our estimates of other income sources). Consequently, if profitability of the cropping system was doubled, incomes from crop production alone would see FDPI's increase from 29% of the poverty line to 58%. Although this is an important reduction in the depth of poverty, it would require almost a quadrupling of FDPI's to ensure incomes from crop production above the poverty line – curtailing the potential of improved farm management practices alone to change the poverty status of farm households.

### 3.2 Income and productivity responses to increasing irrigation frequency with free irrigation

**Fig. 3:**
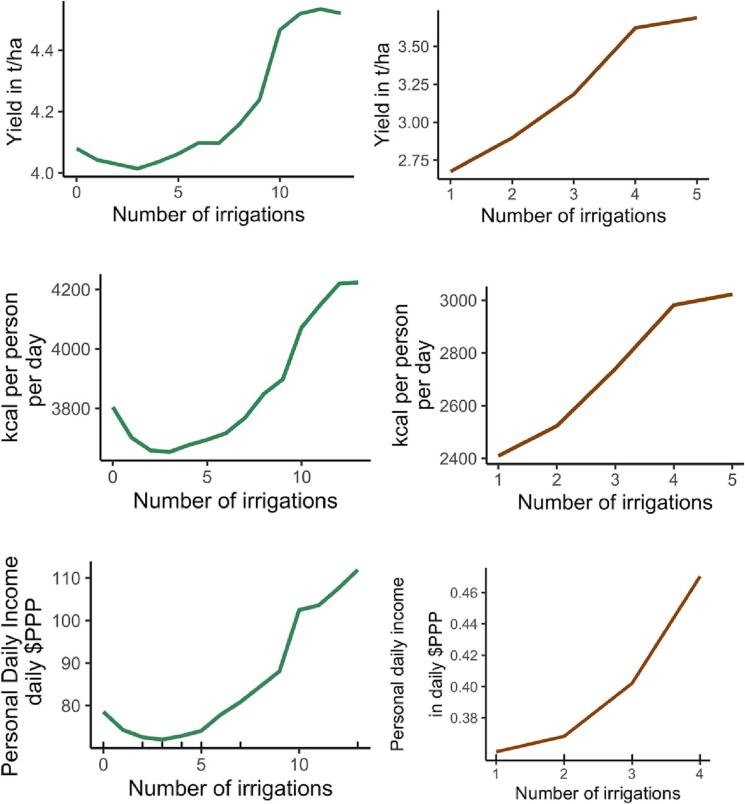
Partial dependency plots from a non-parametric model (randomForest) that was run on a wide set of predictors to estimate, ceteris paribus, the average change in yield (top), daily kcal per person (middle), and FDPI (bottom) with increasing irrigation numbers. Rug (up ticks on the x-axis) indicate data availability and thus the limits of the inference space.

Overall, our data shows that 8% of rice farmers irrigated only once or not at all, 20% irrigated twice, 27% irrigated three times, 16% irrigated four times and 28% irrigated their rice crop five or more times. For wheat, 14% of farmers irrigated once, 50% twice, 30% three times, 5% irrigated four times, and 13 farmers, fewer than 1%, reported irrigating five times. Our results suggest that increasing irrigation frequency is associated with increased yields, but most farmers irrigated at a low frequency (Fig. 3). As expected, the yield response for rice (which is grown during the rainy season) is smaller than for wheat. The average irrigation frequency was 2 irrigations for wheat and 3 for rice. For rice, the difference between mean yields for low and high irrigation-frequency systems was 0.17 t/ ha (see Fig. 3, *p* < 0.01). For wheat, the yield difference between the median low and high irrigation frequency systems was 0.7 t/ha (see Fig. 3, p < 0.01). This positive yield response to increasing irrigation intensity holds true when controlling for other variables and contributing factors (Fig. 3). Although our models do not control for unobserved variables, agronomic studies in these systems have consistently shown that planting dates, fertilizer, varieties, soil types and especially irrigation are consistently the most important predictors for yield (Devkota et al., 2021; McDonald et al., 2022).

Next, we cautiously estimated FDPIs. To make best use of our unique dataset, we focused on key costs of reported fertilizer, and average per ha values for seed, labor and machinery use. For irrigation, we assumed free irrigation first and later approximated the impact of irrigation cost separately with a sensitivity analysis. Given the partial nature of our cost estimates, the true FDPIs are likely lower.

Our results suggest that irrigation-led intensification only has a limited impact on FPDIs in relation to the international poverty line. At low irrigation frequency, the rice-wheat system provided a median 0.36 PPP$ day^−1^, which is 18.9% of the poverty line. The median difference in FDPI between low and high irrigation frequency systems was 0.15 PPP$ day^−1^ for rice and 0.10 PPP$ day^−1^ for wheat. That is a 56% increase in rice and 70% increase in wheat (see Fig. 3, *p* < 0.01). Well-irrigated rice-wheat systems only see median FDPIs of 0.61 PPP$ day^−1^. These increases in FDPI help to close the gap towards the poverty line but remain significantly below 1.90 PPP$ day^−1^. The estimated per ha profits for the 90th percentile – a common definition of attainable yields and profits – of the high-irrigation groups were 3363 PPP$ ha^−1^ for rice and 2397 PPP$ ha^−1^ for wheat. That is a combined 5760 PPP$ ha^−1^. For a median household, that is still a substantial step away from closing the gap to the poverty line for which profits of 9500 PPP$ ha^−1^ are required – and even more so for the 50% of farmers with lower IBI values. The partial dependency plots of the random forest model that control for other variables (Fig. 3) confirm that returns to increasing irrigation frequency in rice-wheat systems are positive but, on average, limited in magnitude. Besides, our per ha yield and profitability results compare well to similar results reported for the improvements of net output from irrigated vs. non-irrigated crop production systems across Asia (Hussain, 2007) – further suggesting that our estimates are in the right order of magnitude and that significantly higher profits are unlikely to materialize.

Lastly, the non-linear shape of the rice response to irrigation (see Fig. 3) cannot be directly explained in this study. Two possible explanations could be that, as indicated in other studies, farmers in low irrigation frequency systems apply irrigation late to save the crop rather than to enhance productivity which may mute the yield response, or that water may not be the only yield-limiting factor in the lower input systems (Urfels et al., 2020). Explaining these aspects requires further research.

### 3.3 Irrigation cost, high-value agriculture, and minimum support price

In the EGP, it is often assumed that poverty alleviation in rural economies is constrained by high irrigation costs and low market prices for agricultural (Shah et al., 2012; Sidhu et al., 2020; Singh, 2018). We explored this assumption but found that farm size remains primary limiting factor for FDPIs from irrigation-led intensification for most rice and wheat farmers. To investigate these claims we first conducted a sensitivity analysis of irrigation costs and looked at the impact of providing all farmers the official minimum support price rather than the farm gate prices they receive (which are substantially lower).

For irrigation, several irrigation technologies with different pricing mechanisms exist in the EGP. This matters because irrigation often comprises the highest component among input costs in the rice-wheat systems of the EGP. High prices are the result of diesel pumps use and expensive rental markets (Shah et al., 2012, Urfels et al., 2020). But options to reduce irrigation cost exist and include better pump selection (Bom et al., 2001; Foster et al., 2019, Urfels et al., 2020) or shifting to electric energy which is expanding quickly in the region.

**Fig. 4:**
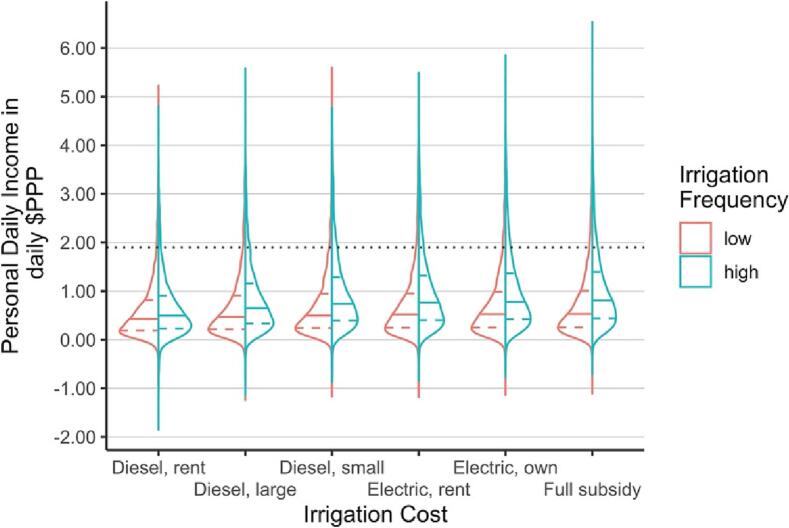
Graphical representation of differences in distribution of combined rice and wheat FDPIs for farmers with low and high irrigation frequencies for different irrigation costs. The violin plots show the distribution of personal daily incomes from full net value of rice-wheat production. Colored, vertical shapes show the density function of each group, the colored and horizontal dashed lines show the 25th and 75th percentile and the solid ones the median. The dotted black horizontal line is the poverty line of 1.90 PPP$ day^−1^.

**Fig. 5:**
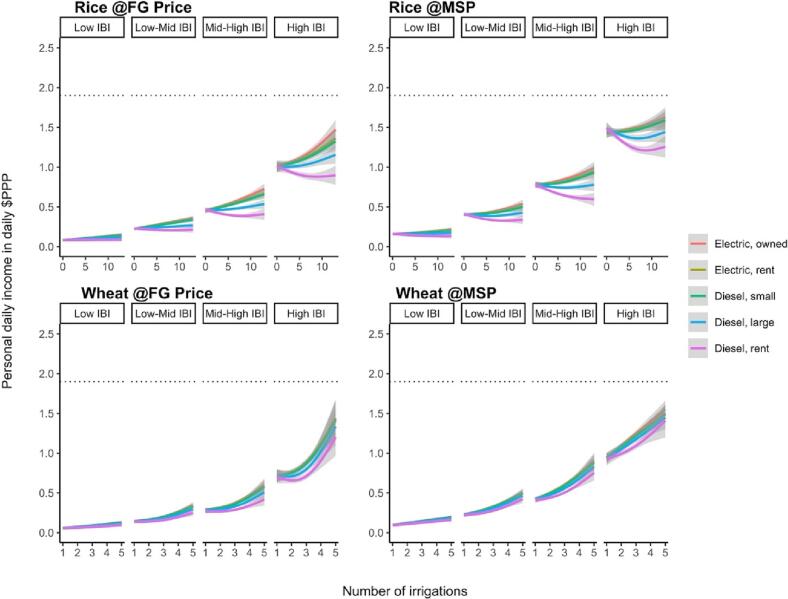
Smoothing splines of estimated average FDPIs in our sample in relation to irrigation intensity, IBI group and irrigation cost. FDPI remains low for low IBI groups irrespective of irrigation intensity or irrigation costs. See Supplementary material for how these average results vary across soil and crop types – key contributing variables – as well as partial effect sizes when controlling for contributing variables. FDPIs only show a strong response for the higher IBI groups. IBI appears to be more consequential for daily incomes than irrigation costs, although the negative slopes indicate that high costs for rented diesel pumps result in profit reductions in rice cultivation. Incomes are derived from full net value of production for rice (top) and wheat (bottom) at the received farm gate prices (left) and most recent minimum support price of 2020 (right). Dashed line is international poverty line of 1.90 PPP$ day^−1^. Non-linear features likely indicate influence of co-variates on yield response (e.g. limiting factors).

On average, the estimated economic returns in the high-irrigation intensity group over the low irrigation group were estimated at 0.11 PPP$ day^−1^ for rental pumps, 0.34 PPP$ day^−1^ for small diesel pumps, and 0.38 PPP$ day^−1^ for electric pumps (Fig. 4, Table 2). But the right-skewed IBI distribution lead to significantly smaller gains for households in the bottom quartile of IBI values (Fig. 5). The median FDPI difference between and high and low irrigation groups for the bottom IBI was 0.14 PPP$ day^−1^ with electric irrigation prices (compared to 1.85 PPP$ day^−1^ for the upper quartile, see Fig. 5).

**Table 2:** Comparing the difference between FDPIs from systems with high and low irrigation frequencies for each irrigation cost group through two-sided paired *t*-test of. All tests are statistically significant (*p* < 0.01). These results indicate the average FDPI difference between farmers with low irrigation frequencies and those with high irrigation frequencies assuming different irrigation costs. This comparison aims to understand the order of magnitude in which irrigation cost, on average, affects FDPIs.

price group	Estimate ($PPP)	statistic	conf. Low ($PPP)	conf. High ($PPP)	method	alternative
Diesel rent	0.09	7.29	0.06	0.11	paired t-test	two.sided
Diesel large	0.24	17.72	0.21	0.26	paired t-test	two.sided
Diesel small	0.36	24.79	0.33	0.38	paired t-test	two.sided
Elec rent	0.35	24.15	0.32	0.37	paired t-test	two.sided
Elec own	0.39	26.41	0.36	0.42	paired t-test	two.sided
Full subsidy	0.40	26.27	0.37	0.43	paired t-test	two.sided

Given that our previous results assumed no irrigation cost, it is not surprising that reductions in irrigation costs do not change our conclusion. But the small impact on farm incomes means that irrigation pricing is unlikely an effective policy lever for affecting irrigation behavior as is often assumed (Sidhu et al., 2020). Our sensitivity analyses indicates that the effect of irrigation prices on FDPIs only becomes of substantial magnitude for the largest farms (Table 2; Fig. 4, Fig. 5) – but not for the majority.

Additional often-cited policy levers formproving farmers' incomes include better market prices such as the minimum support price for cereals or shifting to high-value crops. As with irrigation cost, our data suggests that minimum support prices (see Fig. 5) only have meaningful effects for the highest household IBI quartile. If the official minimum support price for 2020 was paid to farmers in our dataset, the median FPDI for the bottom IBI quartile would amount to a 0.26 PPP$ day^−1^ for low irrigation intensities and 0.30 PPP$ day^−1^ for systems with high irrigation intensities (compared to 2.84 PPP$ day^−1^ and 3.14 PPP$ day^−1^ for the upper IBI quartile). Farmers do see increases in FDPI and thus a reduction in the depth of poverty, but there is no transformative shift above the poverty line if they received the minimum support prices (see Fig. 4).

Another avenue for irrigation to increase FDPIs is by allowing for cultivation of cash crops or triple cropping. As our dataset does not contain such information, we explore these scenarios with profitability values from the literature and a few simple thought experiments: Profitability estimates for diversified or intensified farm systems in the region range from PPP$ 4000 to PPP$ 13,000 per annum with either a third crop (e.g. greengram) and/or significant horticulture integration that replace rice and wheat (Khan and Verma, 2018; Mishra et al., 2021; Sen et al., 2017). These profit margins translate to 0.80–2.60 PPP$ day^−1^ for the median household. In the unlikely event that all farmers in our dataset earned the top line 13,000 PPP$ ha^−1^, 37% of farmers would remain below the poverty line and 15.7% below the 1 PPP$ day^−1^ threshold. This means that highly productive farming systems with well-controlled triple cropping and well-functioning markets may lift a substantial number of farms above the poverty line of 1.90 PPP$ day^−1^ but more than one third remains below it. Besides, the scalability of these diversified systems is limited by biophysical constraints, the food security and cultural value of rice and wheat production, and hinge on market integration, price fluctuations, and farmers' ability to sustain both operation costs and capital investment costs. For many smallholders' diversification thus provides some opportunities for poverty reduction, but with significant risks and investment costs,

These results have consequential implications for irrigation-led intensification policies and programs. First, the economic incentives to invest resources into (irrigation-led) agricultural intensification are rather limited for most farms. Upgrading irrigation infrastructure, for example, into increasingly promoted solar powered irrigation systems with significant upfront investment costs may not provide sufficient returns for most farms (Shah et al., 2018). Low capital investment options such as accessing the expanding and subsidized rural power grid may be more feasible for most farmers. Irrigation systems with high capital investments best target horticulturally oriented farms and farms with high IBI values that have relatively greater incentives as they expect larger returns. Remote and resource-constrained cereal farmers are likely to rely on relatively low-cost, portable, easy to use and repair, diesel pumps until reliable access to electricity has reached these plots. Similarly, finding the right tariff policy for electrified farms – a heavily discussed policy lever – is unlikely to contribute to substantial transformations of rural incomes by raising incomes from crop production (Sidhu et al., 2020). Even with nominal flat tariffs – a pro-poor tariff structure (Sidhu et al., 2020) – our results suggest that farmers in the EGP are not likely to see meaningful changes to their daily incomes from crop production vis a vis the international poverty line.

On a relative basis and in line with the policy goal of doubling farmers' incomes from 2015 to 2016 levels by 2024, the small improvements in income might still be seen as a win for reducing the depth of poverty (Government of India, 2017; Lele, 2019). With low incomes to start with (e.g. our estimated 0.27 PPP$ day^−1^ median FDPI for the bottom IBI quartile), however, it is a long way to go from doubling farm incomes (e.g. to 0.54 PPP$ day^−1^) to transforming farmers' poverty status and reaching 1.90 PPP$ day^−1^. Even impressive gains in agricultural productivity and profitability on a per hectare basis are not going to directly benefit most small farmers but mostly those that are already better off. To benefit the smaller farmers, policymakers need to invest in incremental and coordinated upgrading of agricultural value chains (including irrigation) and job training programs to create inclusive and diversified job opportunities for them.

### 3.4 Home consumption and market participation

Given that transforming the poverty status of most smallholders through better crop production alone is unlikely, this section explores patterns of home consumption and market participation to shed line on food security and off-farm income dimensions. As shown in Section 3.1 (Fig. 2), most smallholder directly consume rice and wheat production at home. One may assume that households with home consumption at sufficiency levels of, e.g., 2700 kcal person^−1^ day^−1^ would increase their incomes by selling additional produce. But multifaceted household, non-household and geographical factors influence smallholders' market participation (Barrett, 2008).

Accordingly, in our dataset, a higher number of irrigations and higher yields were associated with a higher number of kcal that were not sold to markets by the household. Households that had higher IBI values retained an especially high number of kcal person^−1^ day^−1^; ranging from an average of 546 (SD: 279) kcal person^−1^ day^−1^ (for the bottom 25% of IBI) to an average of 2624 (SD: 1277) kcal person^−1^ day^−1^ (for the upper 25% of IBI) in rice and from an average of 531 (SD: 251) kcal person^−1^ day^−1^ (for the bottom 25% of IBI) to an average of 2641 (SD: 1365) kcal person^−1^ day^−1^ (for the upper 25% of IBI) in wheat (not shown). However, most households, including the bottom 25% of IBI values, did sell some rice to markets (see Fig. 2) and the FDPIs from sold produce was double for smallholders with high irrigation frequencies versus ones with low irrigation frequencies: 0.07 PPP$ day^−1^ and 0.15 PPP$ day^−1^. Intra-village insurance and exchanges are likely the source of these variations (Meghir et al., 2019; Townsend, 1994) and larger landowners may increase their consumption while poorer households need to sell produce to meet basic cash needs.

Non-agricultural income in our dataset accounted for at least 50% of household income for most households (Fig. 2) showing that non-agricultural jobs complemented agricultural income streams and might provide resilience enhancing fallback options for climatic and social production shocks (Meghir et al., 2019). These findings are in line with the general notion of non-farm income sources becoming an increasingly important source for household food security (Pingali et al., 2019; Sugden et al., 2014) as the rural economy of the region is currently undergoing rapid structural transformation. And home consumption and in-kind trading are being replaced by increasing commodification, non-farm employment, and purchasing of food crops.

A large literature exists on the dynamics of structural transformations and its effects on the allocation of resources as well as household food security dynamics (Pingali and Sunder, 2017; Tomich et al., 2019; Webb and Block, 2012). Supporting the overall findings of our study, this body of research shows that increases in staple crop productivity and farm income do not necessarily go in hand with positive impact on poverty reductions and food and nutrition security. For example, supply and price levels of non-staple food sources may not keep up with increasing demand. Investing in non-staple value chains and production support is one potential way to increase the benefits of irrigation-led intensification. Doing so helps to maintain low price levels for more diverse diets while large farms improve their production and smaller farmers can seek increasing off-farm employment.

### 3.5 Policy implications and recommendations

In this article, we find that small farm sizes substantially limit the income increases that farmers may gain from raising agricultural productivity through irrigation-led intensification. However, we would like to clearly state two points this article does not claim: First, we focus on systematically assessing the opportunity space and structural limits for increasing farmers' incomes from crop production with a unique large *n* dataset. Secondly, this article does not claim that overall, there are few benefits from irrigation-led intensification of agriculture – we believe that there are many. What we do claim and specify is that the increases in farmers' incomes from crop production are structurally limited by small farm sizes and consequently are likely to remain far below the poverty line for most smallholders in the EGP because the IBI is unlikely to change soon. This claim has important consequences for research and development planning and policies.

Our results show that while irrigation-led intensification is associated with improved productivity of rice-wheat systems, most farms are too small to substantially increase their incomes from crop production through irrigation-led intensification. These findings align with studies of the effects of climate shocks on different farm types and the adoption of conservation agriculture in the region (Keil et al., 2019; Lopez-Ridaura et al., 2018). One possible consequence may be a structural transformation involving many smallholders stepping out of agriculture followed by consolidation of land into larger units (Dorward et al., 2009). However, agricultural development in other land-scarce rice producing countries in Asia, such as Japan or Thailand, did not lead to an increase in farm sizes as it did in Europe or North America. Here, part-time and family farm rice-cultivation with scale-appropriate mechanization has prevailed as a common mode of rice cultivation, albeit with ageing farmers, high levels of subsidies and often inefficient farm management (Doner and Schneider, 2016; Faysse et al., 2020; Veldhuizen et al., 2020). Achieving wider irrigation use and consequently higher levels of productivity therefore requires policymakers to cater to the needs of both small and large farmers with varying investment preferences. These need to consider not only the cost of irrigation but also changes in mobility, off-farm wage rates and opportunities, family labor, and drudgery required to apply water to the fields (Keil et al., 2019; Khatri-Chhetri et al., 2020). Developing an improved understanding of what works where, for whom, and why is required to bring benefits of more reliable irrigation to farmers in the EGP.

For example, better-connected farmers and those with larger landholdings can derive substantial improvements in household incomes from upgrading to solar or grid powered systems. Subsidized solar systems targeted for group use could provide some additional benefits for small farmers that have horticultural plots close to homesteads, with market linkages and transportation infrastructure (Agrawal and Jain, 2019). Many small farmers, however, occasionally rent pumps for irrigation of broadacre plots away from their homesteads (Deininger et al., 2017). For them, electrification or switching to smaller diesel pumps could reduce rental fees, improve production through supplementary irrigation, and perhaps reduce drought risk (our data comes from good rainfall years and better data from drought years is required to test this hypothesis) - thus bolstering food security and climate resilience. But the small profits that can be derived from their small plots and unreliable market linkages are unlikely to contribute to a rural transformation (de Bont et al., 2019; Keil et al., 2019). For small farmers, rice-wheat intensification, and crop production in general may not be the poverty alleviation strategy for the future, as profits are simply too small. Better off-farm income opportunities are required andupgrading agricultural value chains may provide some of them.

Nevertheless, the intensification of rice-wheat farming has a clear role to play in famine prevention among the poor and food security of groups other than smallholder farmers. Therefore, policymakers and practitioners should encourage equitable distribution of irrigation infrastructure and incrementally build a rural knowledge base around sustainable and effective water management at the field level in line with broader sustainable agricultural intensification efforts. Such widespread access to affordable irrigation can help poor households by increasing food production and lowering its price. Cheaper food prices will increase the purchasing power of the poor and reduce the depth of their poverty. However, analysis of this general equilibrium effect of irrigation on poverty reduction is beyond the scope of this paper. Besides, irrigation should not be promoted as a direct way out of poverty for the smallest farms, but policymakers should rather devise options for small farmers to enable supplementary income generation or a move out of agriculture for those who prefer that while using irrigation to buffer their production against climate shocks. Providing honest feedback and telling small farmers that they simply cannot generate sufficient income from farming their land and need to consider other options for generating income may be the best farming advice for them.

Increasing land productivity through irrigation-led intensification of rice-wheat production does not stand at odds with poverty reduction and can contribute to achieving goals such as food security. But achieving both requires a multi-faceted approach that encompasses a focus on farmers' broader livelihood strategies, food security of landless, and strengthening and upgrading agricultural input and output value chains (Hanjra et al., 2009; Namara et al., 2010). Upgrading value chains requires institutional capacity and coordination among line ministries and local governments to foster trust among upstream and downstream stakeholders, avoid technological lock-ins, and invest in critical, reliable infrastructure (Doner and Schneider, 2016; Veldhuizen et al., 2020). Farmers should play a key role in this process. For example, investments in training and education could teach valuable on- and off-farm skills, counteract the notion of agricultural jobs being unattractive, and allow farming households to become more successful farmers. Training programs could also enable off-farm workers and entrepreneurs to support agricultural transformation efforts and help upgrading of rural economies (Hanjra et al., 2009; Ogundari, 2014; Reimers and Klasen, 2013). For instance, supporting the pump and well-drilling sector to incrementally develop a sustainable, equitable, safe, and efficient infrastructure base should be considered as an entry point to create attractive jobs.

Next, sustainability concerns need to be taken more seriously. In the short to mid-term, the EGP faces little risk of groundwater depletion as groundwater recharge is high (>300 mm per year) (Mukherji, 2018; Shah et al., 2018). Aquifers are large and the electrification of groundwater irrigation in West Bengal has not led to any widespread decline in groundwater tables even with intensive Boro rice irrigation (Sarkar, 2020). In the long-term, however, growing water demands from non-agricultural sectors and the impact of increasingly frequent coupled climate shocks imposes additional sustainability concerns and further research is required on the linkages with intensified groundwater use (Raymond et al., 2020). For example, dry spells that are coupled to heatwaves may affect crop growth beyond the sum of individual stresses, and changes in crop choice or new, more resilient cultivars may be required to fulfill food production needs (Kadam et al., 2014). Likewise, the impact of successive droughts and decreased recharge from increasingly erratic rainfall poses further concerns to the sustainability of irrigation in the EGP in the long-term. New methods to assess the impact of climate change on groundwater recharge should inform policy making and diversified and sustainable cropping systems need to be explored (Dillon et al., 2019; Kirby et al., 2016). These sustainability concerns also highlight the importance of climate resilience for ensuringfood security which irrigation-led intensification is likely to substantially contribute to.

Lastly, policy programs should carefully state the main goals of intensifying rice-wheat farming and agricultural intensification in general. It may not transform the poverty status of small farmers, but securely irrigated rice-wheat farming can contribute to food security and build resilience against climate shocks. Strengthening these functions requires a better understanding of where and how to best improve productivity, mindful of farmers' incentives and the complex interactions across bio-physical, socio-economic, and socio-technical factors, their gradients, and their interlinkages with other sustainability outcomes (Molden et al., 2010; Rockstrom et al., 2017; Suhardiman et al., 2018; Zewdie et al., 2020). Several factors interact to shape appropriate irrigation use: soil and drainage types of the plots, weather conditions, crop varieties, crop types and the timeliness and amount of irrigation. At the same time, social and technical requirements for the use of different irrigation systems add another layer of complexity and can constrain irrigation use (Westling et al., 2019). Building farmers' knowledge and experience are crucial for navigating and managing these factors at the plot level. Future research needs to develop solutions that appreciate these dynamics across large landscapes and can pinpoint potential avenues for increasing land productivity and system profitability that are anchored in context- and place-specific development trajectories and informed by patterns of spatial inter-village and intra-village heterogeneity (Lambe et al., 2020). Expanding irrigation features high on the political agenda, and a new wave of irrigation research is needed for effectively utilizing irrigation infrastructure and fostering targeted and systemic improvement in a rapidly changing food system.

## 4 Conclusions

We investigated how irrigation-led intensification of rice-wheat systems may impact personal daily incomes derived from crop production of smallholder farmers in the Eastern Gangetic Plains. We find that crop production alone may not lift most household above the poverty line as most farms are too small to generate substantial incomes from crop production – especially from cereals. Nevertheless, irrigation reduces the depth of poverty and provides substantial benefits for agricultural productivity that play a key role in preventing famine and strengthening food security and climate resilience. Therefore, we argue that irrigation development should be considered as part of cross-sectoral efforts and coordinated upgrading of the rural economy that creates both agricultural and non-agricultural jobs and skills. As such, policymakers should develop targeted investments that can support irrigation-led agricultural intensification where it is likely to produce the largest payoffs for food security and climate resilience.

Our study is limited due to incomplete information on production costs and a more detailed analyses focusing on causal inference is needed in the future to provide more granular insights into the relationships between irrigation and poverty in the EGP. Such an analysis, especially if of spatial nature, can further inform regional investment decisions and targeting. Likewise, indirect effects of irrigation investments on poverty – such as through off-farm job generation – also require additional research.

## Declaration of Competing Interest

The authors declare that they have no conflict of interest.

## Data Availability

Data will be made available on request.

## Appendix A Supplementary data

Supplementary material Image 1
