# Supplementary material for: Farm size limits agriculture's poverty reduction potential in Eastern India even with irrigation-led intensification
Source: Agric Syst. 2023 Apr;207:103618. doi: 10.1016/j.agsy.2023.103618 (PMC10114281; doi:10.1016/j.agsy.2023.103618)
Supplement: Supplementary file 1 — Supplementary material [file mmc1.docx]

Supplementary materials

**Farm size limits agriculture’s poverty reduction potential in Eastern India even with irrigation-led intensification**

Anton Urfels^a,b,c,*^, Kai Mausch^d^, Dave Harris^e,f^, Andrew McDonald^g^, Avinash Kishore^h^, Balwinder-Singh^i^, Gerardo van Halsema^b^, Paul Struik^c^, Peter Craufurd^a^, Timothy Foster^k^, Vartika Singh^h^, Timothy Krupnik^l^

**
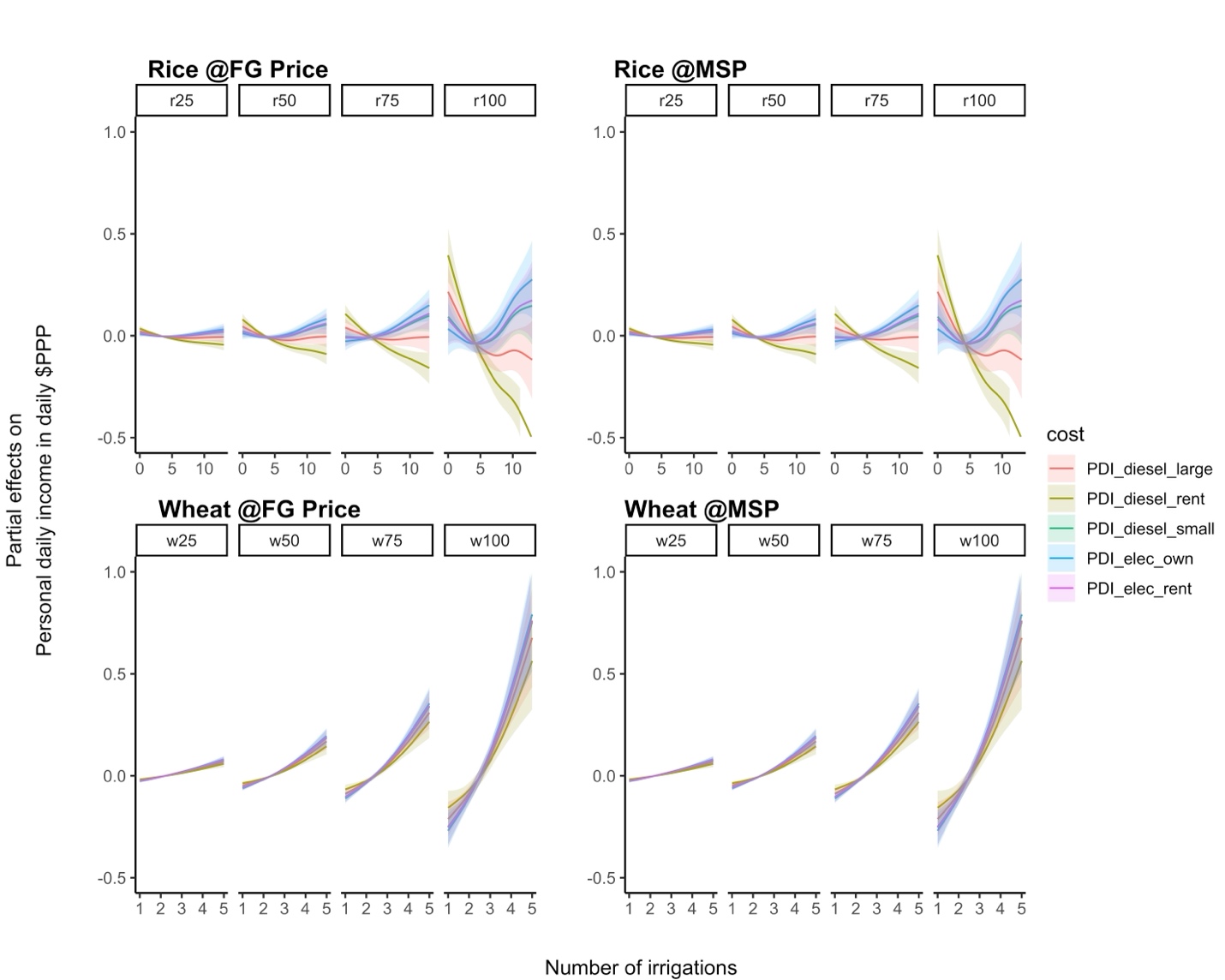
**

**Supplementary Figure 1.** GAM models showing estimated partial effects of irrigation numbers on FDPIs in our sample by IBI group and irrigation cost. FDPI effects remain low for low IBI groups irrespective of irrigation intensity or irrigation costs. FDPIs only show a strong response for the higher IBI groups. IBI appears to be more consequential for FDPIs than irrigation costs, although the figure indicates that high costs for rented diesel pumps result in profit reductions in rice cultivation. Incomes are derived from full net value of production for rice (top) and wheat (bottom) at the received farm gate prices (left) and most recent minimum support price of 2020 (right). Negative slopes indicate that irrigation is not profitable with the respective cost of irrigation of the associated irrigation technology. Models were run with the mgcv package for R with the method parameter set to “REML” and irrigation number, farm gate price, planting date, agricultural income share, urea rate, cultivated area as smoothing splines and soil class, crop type, and a binary pest incidence variable as ordinary independent variables.


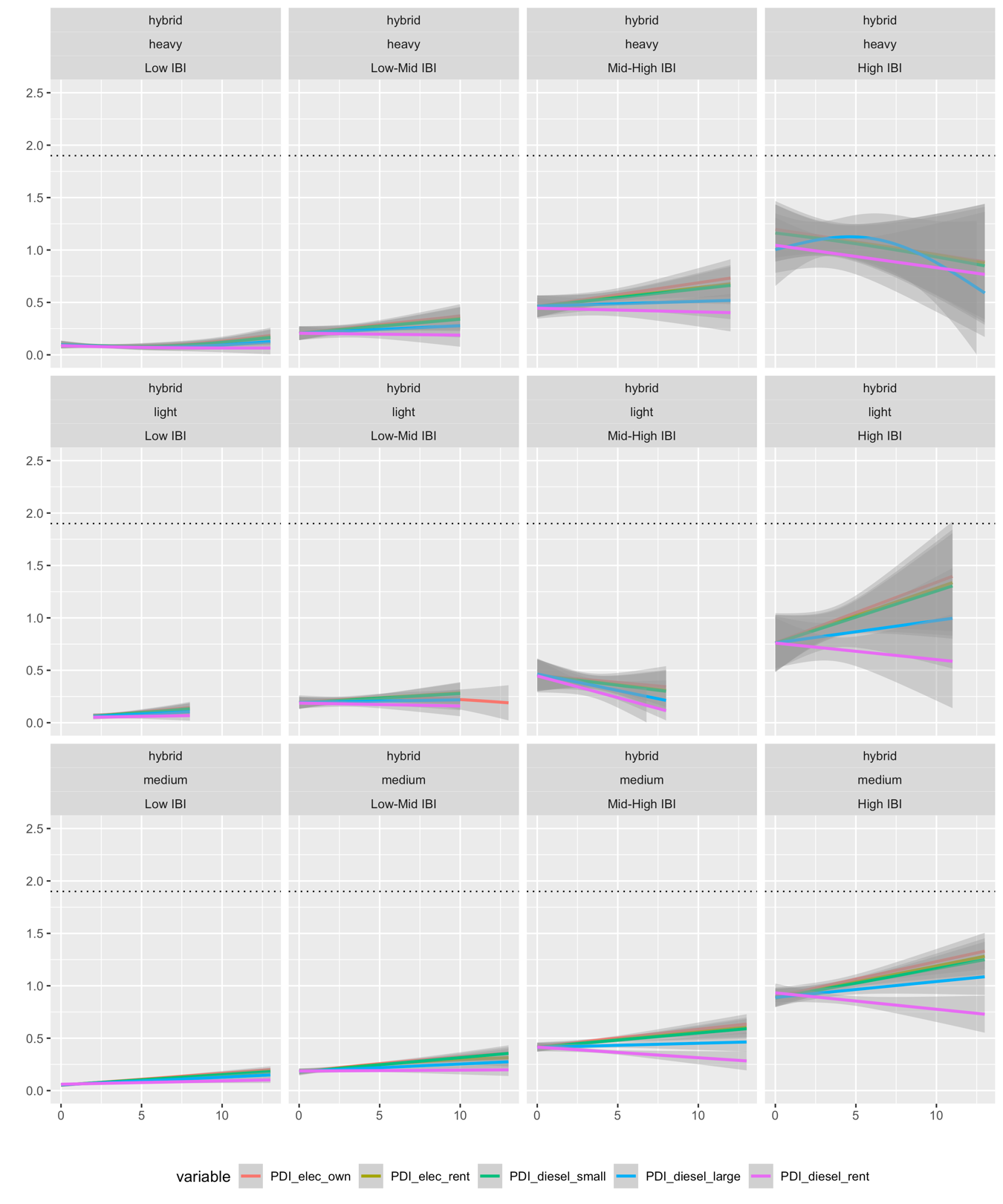


**
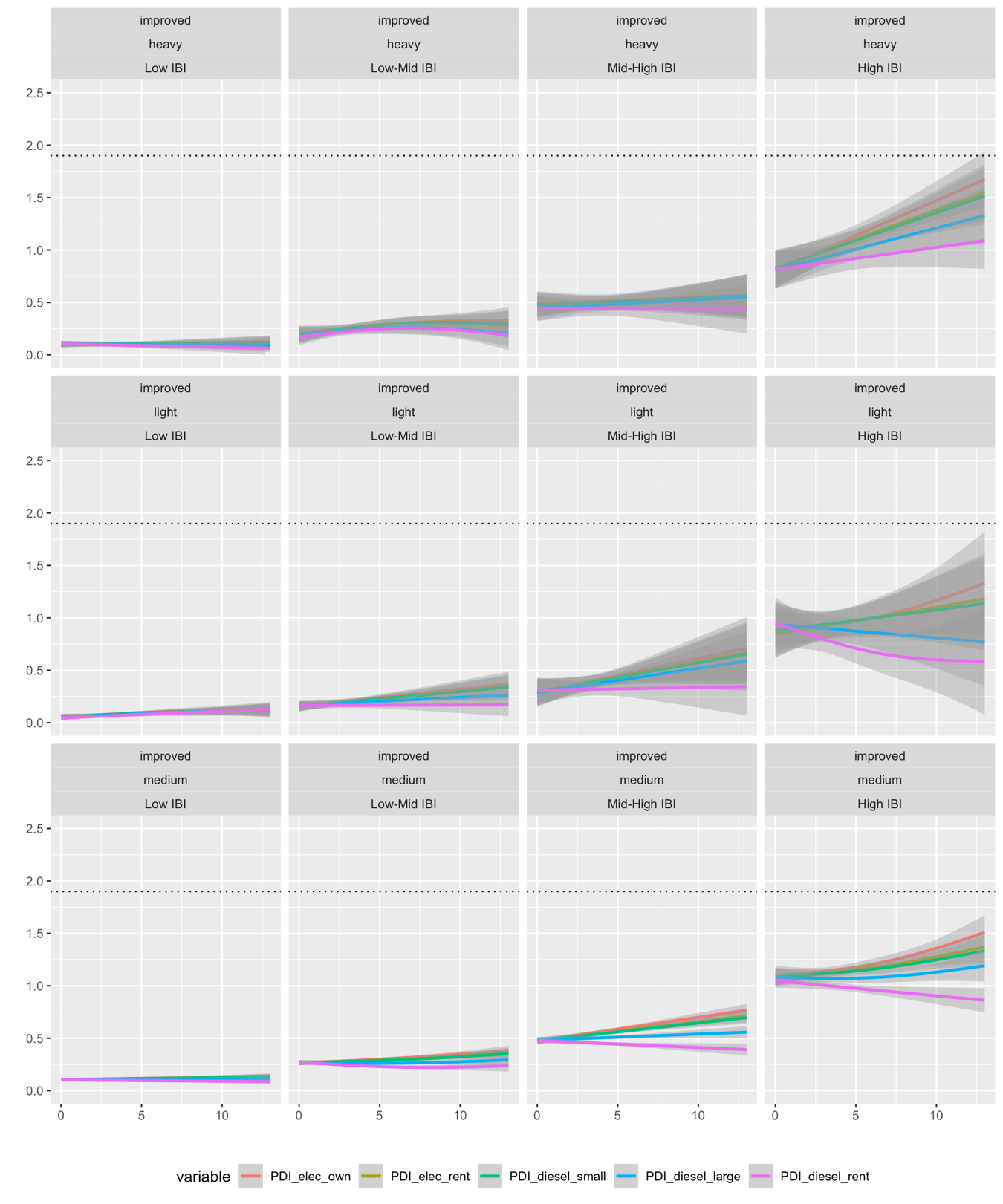
**

**
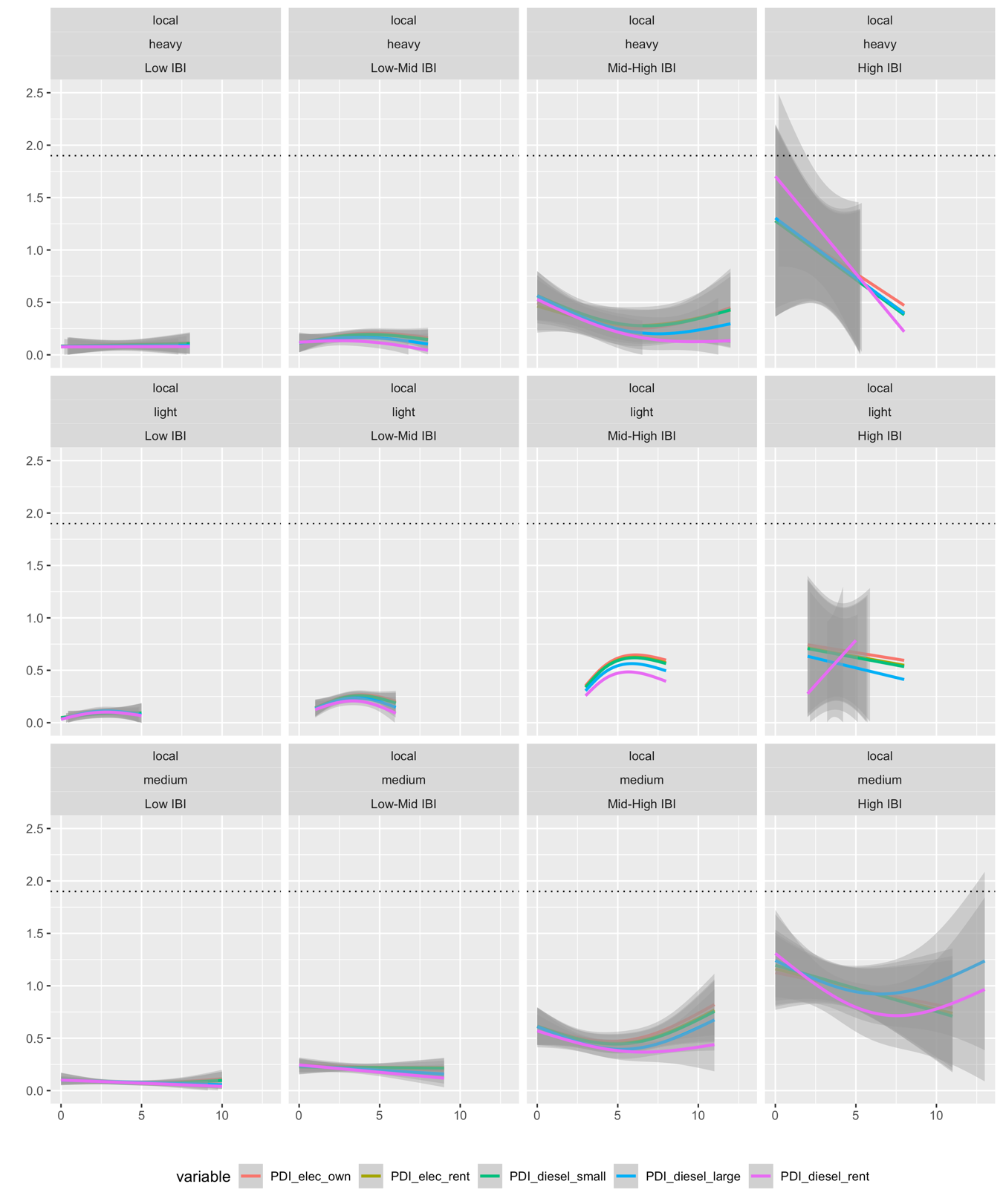
**

**Supplementary Figure 2.** Smoothing splines for rice showing estimated average FDPIs associated with IBI quartiles, irrigation intensity, and irrigation costs for each combination of soil classes (light, medium, heavy) and crop variety types (local, improved, hybrid) included. Some anomalies in the graphs result from insufficient data on a few crop types – soil type groups. Nevertheless, the results show that IBI is the overriding factor affecting FDPIs even with increasing irrigation intensity, regardless of soil classes and crop types.


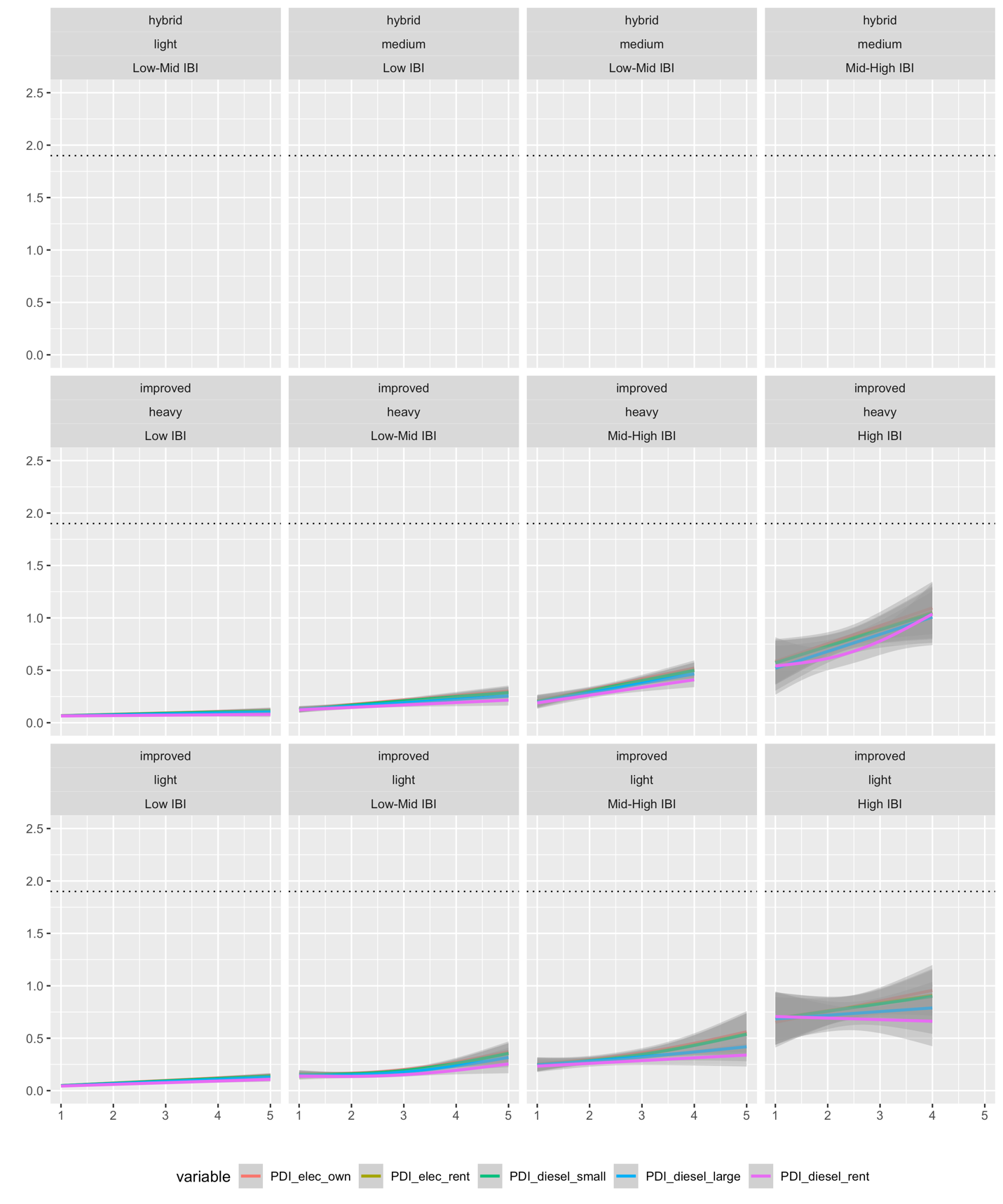


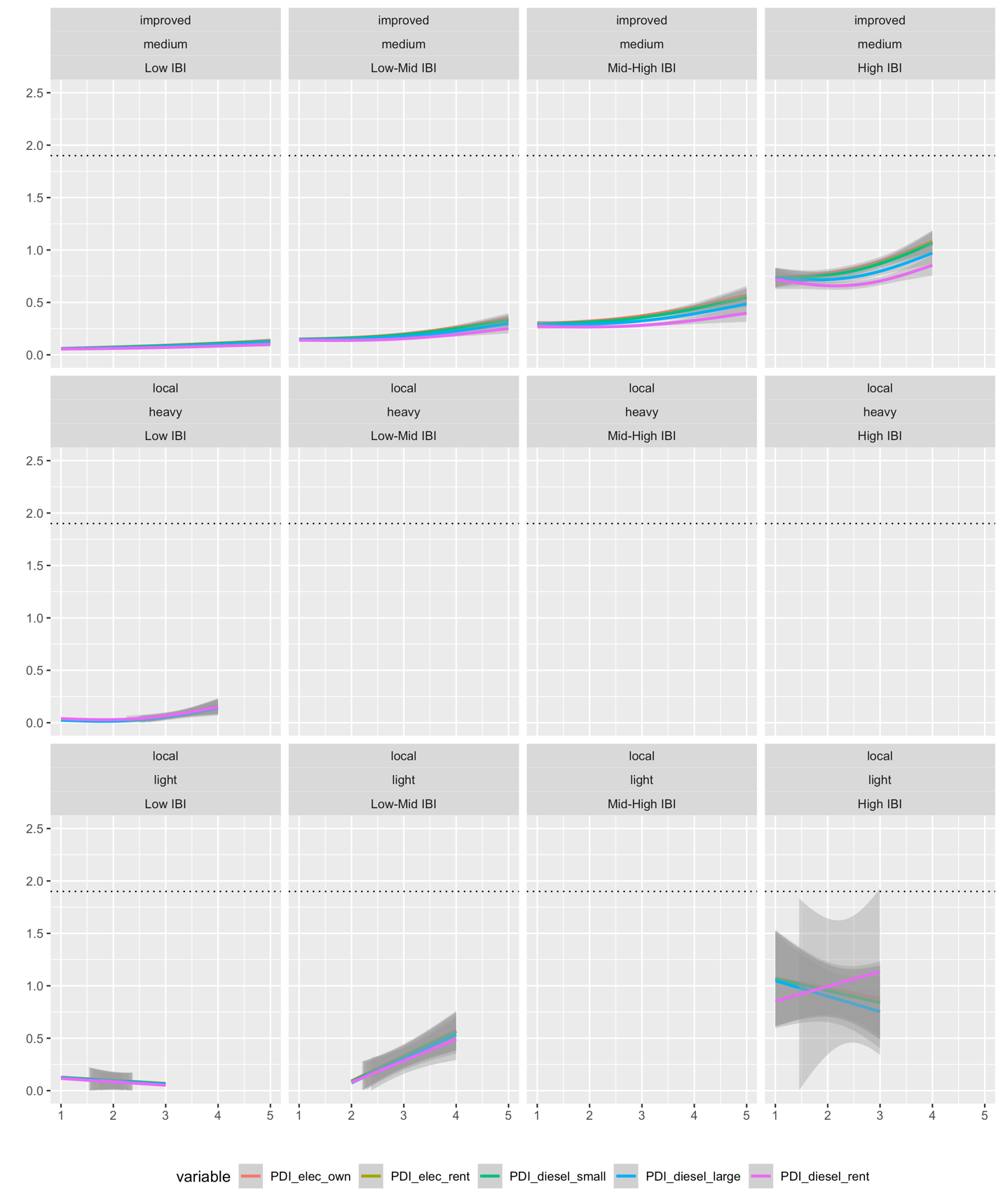


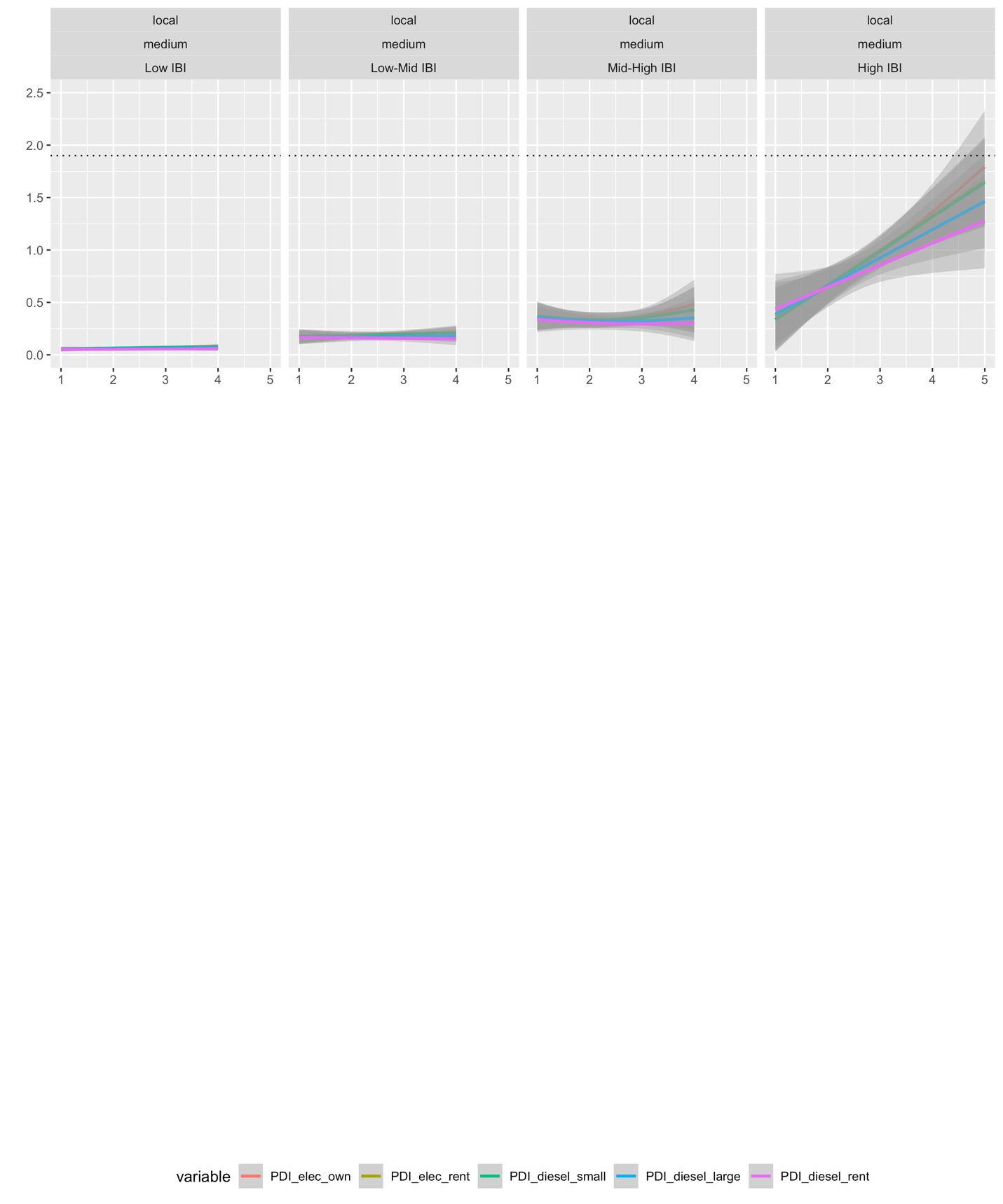


**Supplementary Figure 3.** Smoothing splines for wheat showing estimated average FDPIs associated with IBI quartiles, irrigation intensity, and irrigation costs for each combination of soil classes and crop variety types included. Some anomalies in the graphs result from insufficient data on a few crop types – soil type groups. Nevertheless, the results show that IBI is the overriding factor affecting FDPIs even with increasing irrigation intensity, regardless of soil classes and crop types.
